# Human fetal and adult epicardial-derived cells: a novel model to study their activation

**DOI:** 10.1186/s13287-016-0434-9

**Published:** 2016-11-29

**Authors:** Asja T. Moerkamp, Kirsten Lodder, Tessa van Herwaarden, Esther Dronkers, Calinda K. E. Dingenouts, Fredrik C. Tengström, Thomas J. van Brakel, Marie-José Goumans, Anke M. Smits

**Affiliations:** 1 Department of Molecular Cell Biology, Leiden University Medical Center, P.O Box 9600, Postzone S-1-P, 2300RC Leiden, The Netherlands; 2 Department of Cardiothoracic Surgery, Leiden University Medical Center, P.O Box 9600, Postzone S-1-P, 2300RC Leiden, The Netherlands

**Keywords:** Epicardium, In vitro model, Epithelial-to-mesenchymal transition, Human EPDCs, Cardiac development, TGFβ

## Abstract

**Background:**

The epicardium, a cell layer covering the heart, plays an important role during cardiogenesis providing cardiovascular cell types and instructive signals, but becomes quiescent during adulthood. Upon cardiac injury the epicardium is activated, which includes induction of a developmental gene program, epithelial-to-mesenchymal transition (EMT) and migration. However, the response of the adult epicardium is suboptimal compared to the active contribution of the fetal epicardium to heart development. To understand the therapeutic value of epicardial-derived cells (EPDCs), a direct comparison of fetal and adult sources is paramount. Such analysis has been hampered by the lack of appropriate culture systems.

**Methods:**

Human fetal and adult EPDCs were isolated from cardiac specimens obtained after informed consent. EPDCs were cultured in the presence of an inhibitor of the TGFβ receptor ALK5. EMT was induced by stimulation with 1 ng/ml TGFβ. PCR, immunofluorescent staining, scratch assay, tube formation assay and RT^2^-PCR for human EMT genes were performed to functionally characterize and compare fetal and adult EPDCs.

**Results:**

In this study, a novel protocol is presented that allows efficient isolation of human EPDCs from fetal and adult heart tissue. In vitro, EPDCs maintain epithelial characteristics and undergo EMT upon TGFβ stimulation. Although similar in several aspects, we observed important differences between fetal and adult EPDCs. Fetal and adult cells display equal migration abilities in their epithelial state. However, while TGFβ stimulation enhanced adult EPDC migration, it resulted in a reduced migration in fetal EPDCs. Matrigel assays revealed the ability of adult EPDCs to form tube-like structures, which was absent in fetal cells. Furthermore, we observed that fetal cells progress through EMT faster and undergo spontaneous EMT when TGFβ signaling is not suppressed, indicating that fetal EPDCs more rapidly respond to environmental changes.

**Conclusions:**

Our data suggest that fetal and adult EPDCs are in a different state of activation and that their phenotypic plasticity is determined by this activation state. This culture system allows us to establish the cues that determine epicardial activation, behavior, and plasticity and thereby optimize the adult response post-injury.

**Electronic supplementary material:**

The online version of this article (doi:10.1186/s13287-016-0434-9) contains supplementary material, which is available to authorized users.

## Background

Although the epicardium is merely a cell layer covering the myocardium, it is increasingly gaining interest due to its contribution to cardiac development, as well as its potential role in cardiac repair.

The epicardium derives from the proepicardial organ, a transient structure located at the base of the heart from where cells migrate to ultimately envelop the developing myocardium. Subsequently, a subset of epicardial cells undergo epithelial-to-mesenchymal transition (EMT), thereby forming epicardial-derived cells (EPDCs) that migrate into the subepicardial space [1, 2]. EPDCs impact on heart development by differentiating into several cardiac cell lineages, including fibroblasts, vascular smooth muscle cells (reviewed in [3]) and, potentially into coronary endothelial cells and cardiomyocytes [4–6]. Moreover, the remaining fetal epicardial layer produces growth factors and cytokines that support the growth of the underlying myocardium [7]. The importance of the epicardium in heart development is underscored by the finding that disrupting its proper formation results in a hypoplastic myocardium [8–10]. Furthermore, blocking epicardial migration into the myocardium during development leads to dysmorphic hearts, including absence of the apex and reduction of myocardial thickness [11].

In contrast, in the healthy adult heart, the epicardium is a quiescent layer. However following cardiac injury, epicardial cells are re-activated, which includes upregulation of a developmental gene program, including the embryonic epicardial genes Wilms’ tumor 1 (*Wt1*) and T box 18 (*Tbx18*) [12–14]. Subsequently, as part of their cellular activation, the epicardial layer expands and cells undergo EMT upon which they migrate into the underlying tissue. Here they contribute to the endogenous cardiac repair mechanisms including scar formation, and possibly cardiac regeneration (reviewed in [15, 16]). The significance of epicardial activation in the adult is shown by the fact that preventing EMT and epicardial expansion post-injury leads to impaired cardiac function [17]. Conversely, when the heart is primed via intraperitoneal injection of Thymosin β4 prior to injury, epicardial activation is increased. This resulted in an improved epicardial response, enhanced cardiac output and reduced infarct size [13]. Altogether this shows the importance of epicardial activation and the important role the epicardium has and could have as a myocardial support mechanism following injury.

Given the activation of a developmental program upon injury, a thorough understanding of fetal EPDC behavior is crucial to appreciate the epicardial potential in cardiac repair. To date the intrinsic behavior of fetal and adult EPDCs has not been compared. Such comparison could provide insight into the factors and pathways that determine the optimal epicardial response observed in cardiac development. This knowledge could then be applied to the adult epicardium to enhance the epicardial activation response post-injury. To this end, in vitro models would be indispensable for epicardial research. However, one of the major obstacles has been the inability to consistently isolate a pure population of EPDCs from both fetal and adult heart tissue, and maintain these cells in a stable epithelial state in vitro*.*

In the present study, we describe a novel method for the isolation and expansion of EPDCs derived from fetal and adult human heart tissue. We show that cells from both sources can maintain an epicardial phenotype in culture, and participate in several processes related to in vivo EPDC behavior, including EMT and migration. Although similar in many aspects, our culture model revealed that there are important differences between fetal and adult EPDCs. These differences are likely related to our observation that fetal EPDCs respond faster to EMT-inducing cues. Therefore, we propose that fetal and adult EPDCs are present in different stages of activation. Our culture system will prove to be a useful tool to investigate the process of activation in both cell sources and therefore open new avenues in understanding the full epicardial potential for cardiac repair.

## Methods

### Human cardiac tissue collection

Human fetal hearts, aged between 10 to 22 weeks post gestation, were collected after elective abortion without medical indication and based on individual informed consent. Adult human atrial samples (auricles) were obtained during cardiac surgery. These specimens are obtained as redundant material during right atrial cannulation for extracorporeal bypass and were anonymously collected as surgical waste under general informed consent. Experiments were performed using both male and female samples and were carried out according to the official guidelines of the Leiden University Medical Center and approved by the local Medical Ethics Committee. This research conforms to the Declaration of Helsinki.

### Isolation and culture conditions of human EPDCs

Fetal and adult EPDCs were isolated by separating the epicardium from the underlying myocardium. The tissue was processed into small pieces and digested during three rounds of Trypsin 0.25%/EDTA incubation (1:1; Serva and USH products) for a total of 30 minutes at 37 °C. The suspension was subsequently passed through a series of syringes of decreasing size (19G to 22G). The cell suspension was passed through a 100-μm cell strainer (BD Falcon, Franklin Lakes, NJ, USA), collected and plated on 0.1% gelatin-coated dishes (Sigma-Aldrich, St. Louis, MO, USA). The established EPDCs were cultured in a mixture of 1:1 Dulbecco’s modified Eagle’s medium (DMEM-glucose low; Invitrogen, Carlsbad, CA, USA) and Medium 199 (M199; Invitrogen) supplemented with 10% heat-inactivated fetal calf serum (FCS; Gibco, Carlsbad, CA, USA), and 100 U/ml penicillin/streptomycin (Gibco). To maintain cells in an epithelial state, the ALK5-kinase inhibitor SB431542 (5–10 μm; Tocris Bioscience, Ellisville, MO, USA) was added. In experiments aimed at inducing EMT, cells were stimulated with 1 ng/ml transforming growth factor beta 3 (TGFβ3), without SB, for 4 days. EPDCs used in experiments did not exceed ten passages.

### mRNA isolation and quantitative RT-PCR

Total RNA was isolated using Tripure (Roche, Basel, Switzerland) according to the manufacturer’s protocol. Samples were treated with recombinant DNAseI (Roche) followed by cDNA synthesis using the Revert Aid H minus First Strand Synthesis Kit with OligodT primers (Thermo Fisher Scientific, Waltham, MA, USA); both according to the manufacturers’ recommendations. Subsequently, qRT-PCR was performed using SYBR Green (Bio-Rad Laboratories, Hercules, CA, USA) and run on a CFX Connect Real-Time System (Bio-Rad). For visualization purposes, samples were run for 35 cycles and processed on a 3% agarose gel. Primer sequences are available upon request.

### EMT PCR array

Fetal and adult cell isolations (three independent isolations for each source) were cultured in either SB431542, TGFβ3 or empty medium for 4 days. RNA was isolated using the NucleoSpin® columns (Macherey-Nagel, Düren, Germany) according to the manufacturer’s instructions. Subsequently, cDNA was synthesized using the RT^2^ First Strand Kit (SA Biosciences, Valencia, CA, USA). A RT^2^ profiler array was performed for EMT-related genes (type PAHS-090Z from SA Biosciences) using SYBR® Green qPCR Mastermix (SA Biosciences). Hierarchical cluster analysis was performed using Matlab (The MathWorks, Inc., Natick, MA, USA; version 2016a).

### Immunohistochemistry

Cells were fixed in 4% paraformaldehyde and permeabilized using PBS/0.25% Triton X100. Samples were blocked in 5% FCS in PBS or 10% FCS/0.1% Triton X-100 in PBS for 15 minutes and subsequently incubated overnight at 4 °C with primary antibody. Antibodies were diluted 1:100 in blocking buffer and directed against WT1 (Abcam, Cambridge, MA, USA), TBX18 (Sigma-Aldrich), TCF21 (Santa Cruz Technologies, Dallas, TX, USA), ZO-1 (Life Technologies, Carlsbad, CA, USA), αSMA (Sigma-Aldrich), PECAM1 (Santa Cruz Technologies) and vimentin (Cell Signaling, Danvers, MA, USA). Alexa Fluor® 488- and 555-conjugated secondary antibodies (Invitrogen) were used at a 1:250 dilution. Phalloidin, conjugated to Alexa Fluor® 488, was applied at a 1:250 dilution. Slides were mounted with Prolong Gold containing DAPI (Invitrogen). All pictures were handled in an equal fashion.

### Scratch assay

Scratch assays were performed by plating EPDCs in 48-well plates (in growth medium). Cells were grown to a nearly confluent monolayer, and subsequently cultured for 48 hours in the presence or absence of SB431542, or stimulated with 1 ng/ml TGFβ3 to induce partial EMT. The scratch was created as previously described [18] without changing medium. Cells were subsequently monitored for 12 hours, taking pictures every 4 hours. The percentage gap closure was measured using Matlab (version 2016a).

### Matrigel assay

Five thousand EPDCs were resuspended in growth medium containing 25 ng/ml VEGF (Peprotech, Rocky Hill, NJ, USA) and seeded onto Matrigel™-coated (BD Bioscience, San Diego, CA, USA) 96-well plates. After 24 hours the formation of tubes was measured using the Angiogenesis Analyzer plugin for ImageJ (developed by Gilles Carpentier).

### Statistics

Graphs are represented as mean ± SD of at least three independent experiments (unless otherwise indicated). Fetal and adult samples were compared using an unpaired Student’s *t* test. A paired Student’s *t* test was used when different stimulations were applied to the same EPDC culture. Significance was assumed when *p* < 0.05. GraphPad Prism (GraphPad Software, San Diego, CA, USA; version 6) was used for statistical analysis.

## Results

### Fetal and adult human EPDCs maintain their epicardial character in vitro

The epicardium is located directly on the myocardium and can be separated from the underlying tissue using forceps (Additional file 1: Figure S1a). We confirmed the epicardial identity of the isolated layer by qRT-PCR. The epicardial marker *WT1* was expressed, while no expression of the mesenchymal marker alpha-smooth muscle actin (α*SMA*) or the cardiomyocyte markers beta-myosin heavy chain (β*MHC*), cardiac troponin T (*cTnT*) and cardiac-actin (*cActin*) was observed (Additional file 1: Figure S1b and data not shown). This validated that we exclusively isolated the epicardium.

**Fig. 1:**
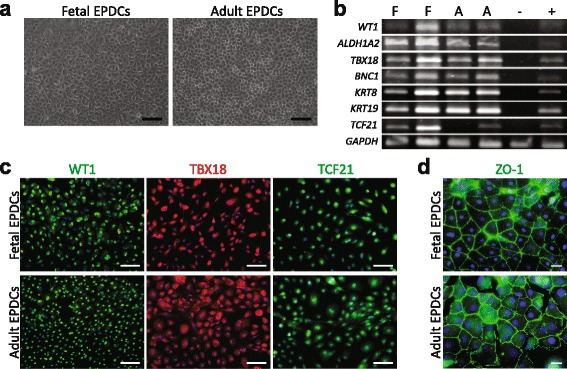
Human fetal and adult EPDCs express epicardial markers in culture. **a** Isolated fetal and adult EPDCs displayed a cobblestone appearance indicative of their epithelial phenotype (scale bar: 100 μm). **b** The presence of several epicardial markers was confirmed by PCR. Shown are two independently isolated fetal (*F*) and adult (*A*) EPDC cultures. Total adult myocardium, without epicardium, was used as negative control (-) and non-cultured epicardium as positive control (+). **c** Immunofluorescent staining showed that both EPDC populations were positive for the transcription factors WT1, TBX18 and TCF21 (scale bars: 100 μm). **d** ZO-1 depicted the tight junctions substantiating their epithelial phenotype (scale bar: 20 μm). *EPDCs* epicardial-derived cells

Primary cell cultures of both fetal and adult EPDCs were established by generating a single-cell suspension from the epicardial layer. The cells were cultured in medium containing SB431542 (SB) which is a selective inhibitor of the activin receptor-like kinase (ALK5), a type I receptor of transforming growth factor beta (TGFβ). Using this approach, isolated EPDCs could be maintained and expanded in culture as a monolayer of epithelial cells, while maintaining their characteristic cobblestone morphology (Fig. 1a).

To determine if cultured human EPDCs maintained their epicardial signature in vitro, their gene expression pattern was assessed. The common epicardial marker transcripts *WT1*, *TBX18*, aldehyde dehydrogenase 1 family member A2 (*ALDH1A2*) and transcription factor 21 (*TCF21*) [11, 12, 19–21] were present in both fetal and adult EPDCs as shown in Fig. 1b. In addition, several other epicardial genes, like basonuclin 1 (*BNC1*) and keratin 8 (*KRT8*), that were found enriched in the developing epicardium [22], were present in both populations (Fig. 1b and Additional file 1: Figure S2a). Furthermore, transcripts that were previously reported to be present in EPDCs or epithelial cells [22–28] were analyzed and found to be present. Their expression is summarized in Additional file 1: Figure S2a. Immunofluorescent analysis confirmed the presence (Fig. 1c) and nuclear localization (Additional file 1: Figure S2b-d) of the proteins and epicardial transcription factors WT1, TBX18, and TCF21. Moreover, the isolated fetal and adult EPDCs formed tight junctions, demonstrated by the presence of zona occludens-1 (ZO-1) at their cell borders (Fig. 1d).

Taken together, these data show that the epicardial layer can be successfully isolated from human fetal and adult heart tissue. The derived EPDCs are of epicardial origin, and maintain their epicardial characteristics in culture.

### Cultured EPDCs do not display markers of other heart-resident cell types

**Fig. 2:**
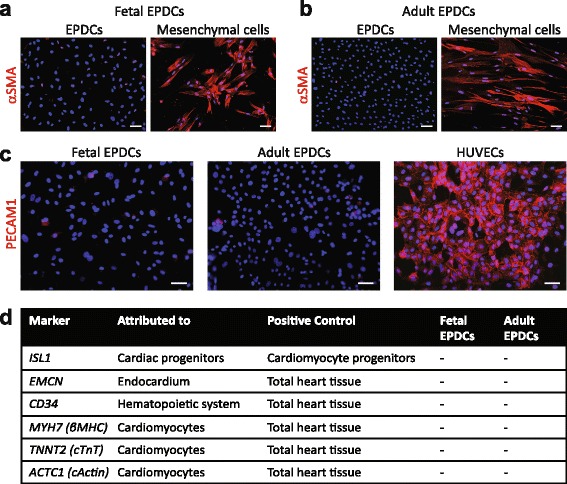
Cultured EPDCs do not display markers of other heart-resident cell types. Immunofluorescent staining confirmed the absence of αSMA in both (**a**) fetal and (**b**) adult EPDCs, using mesenchymal cells as positive control (scale bar: 50 μm). **c** EPDCs were negative for the endothelial marker PECAM1 (scale bar: 50 μm). Human umbilical vein endothelial cells (HUVECs) were used as positive control. **d** qRT-PCR analysis confirmed the absence of markers attributed to cardiac progenitor cells, the endocardium, hematopoietic system, and myocardium. *EPDCs* epicardial-derived cells

To verify further that we indeed obtained human epicardial cells, we aimed to exclude potential contamination of our cultures with other cardiac cell types. Both fetal (Fig. 2a) and adult derived cells (Fig. 2b) did not express *αSMA*. In addition, expression of the mesenchymal transcript periostin (*POSTN*) was low (Additional file 1: Figure S3a-b). Since endothelial cells have a comparable cobblestone phenotype, we examined the expression of platelet-endothelial cell adhesion molecule 1 (PECAM1) by immunofluorescent analysis, and found it to be absent (Fig. 2c). qRT-PCR corroborated the absence of *PECAM1* and VE-cadherin (*CDH5*; Additional file 1: Figure S3c). Furthermore, mRNAs encoding markers attributed to the endocardium, cardiac progenitor cells, hematopoietic system, and cardiomyocytes were not present (Fig. 2d and Additional file 1: Figure S3d–g).

Overall, we show that both fetal and adult EPDCs could be distinguished from the myocardial and endocardial lineage as well as from other heart-resident cell types.

### Fetal and adult human EPDCs undergo EMT upon TGFβ stimulation

EMT is fundamental to formation of the heart during development and repair following injury [29]. EMT is a gradual process during which epithelial cells lose cell-cell contact and their epithelial morphology changes into a mesenchymal elongated phenotype. TGFβ has been shown to play a pivotal role in inducing EMT in for example proepicardial cells [30] and human adult EPDCs leading to, among others, downregulation of *WT1* [27].

**Fig. 3:**
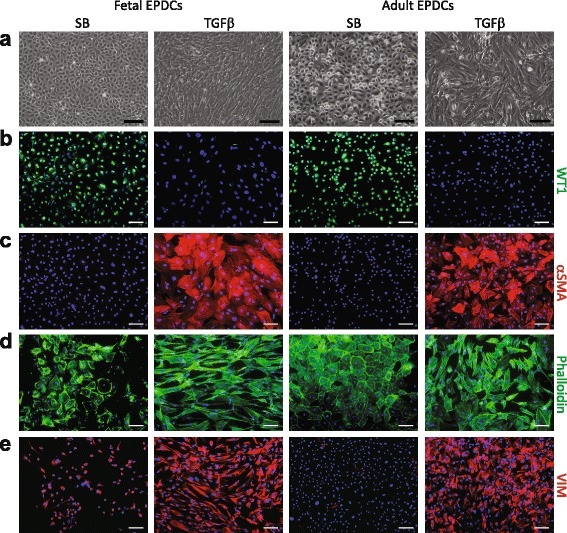
Human fetal and adult EPDCs undergo EMT upon TGFβ stimulation. **a** Phase contrast microscopy showed that SB-treated cells displayed a characteristic cobblestone morphology, while upon TGFβ stimulation both fetal and adult EPDCs underwent EMT. This was evident by transformation into a spindle-shaped and elongated morphology. Change in morphology was accompanied by (**b**) a decrease in nuclear WT1 and (**c**) an increase in αSMA. Upon TGFβ stimulation, (**d**) phalloidin visualized the increase in actin filaments and (**e**) VIM staining showed an organized network of intermediate filaments (scale bars: 100 μm). *EPDCs* epicardial-derived cells, *SB* medium containing SB431542, *TGFβ* transforming growth factor beta

We compared the ability of fetal and adult EPDCs to respond to TGFβ and undergo EMT. Four days after TGFβ stimulation, both fetal and adult EPDCs lost their characteristic cobblestone morphology and transformed into elongated spindle-shaped cells, indicators of a mesenchymal phenotype (Fig. 3a). Besides a morphological change, we confirmed the occurrence of EMT by immunofluorescent analysis and qRT-PCR. A decrease in nuclear WT1 expression levels in both fetal and adult cells was observed (Fig. 3b), as well as an increase in αSMA expression (Fig. 3c). In addition, in EPDCs phalloidin-labeled F-actin was predominantly organized in cortical bundles located at the cell surface, while this expression pattern changed into stress fibers that traversed the cells upon TGFβ stimulation (Fig. 3d). Concurrently, the mesenchymal marker vimentin (VIM) showed an increase in organized networks of intermediate filaments after stimulation (Fig. 3e). A decline in mRNA expression of *ALDH1A2* and the epithelial marker E-cadherin (*CDH1*) was observed in fetal and adult EPDCs (Additional file 1: Figure S4a-b). In addition, upregulation of *POSTN* and fibronectin 1 (*FN1*) illustrated their transition toward a mesenchymal cell fate (Additional file 1: Figure S4c-d). In summary, these data show that fetal and adult EPDCs have the ability to undergo EMT upon TGFβ stimulation.

### Fetal and adult EPDCs have a different migration ability upon TGFβ stimulation

In vivo, both during development as well as after injury, induction of EMT in epicardial cells is followed by migration of these cells into the underlying tissue. To determine whether cultured fetal and adult EPDCs retained this ability, we assessed their functional migration by performing scratch assays.

**Fig. 4:**
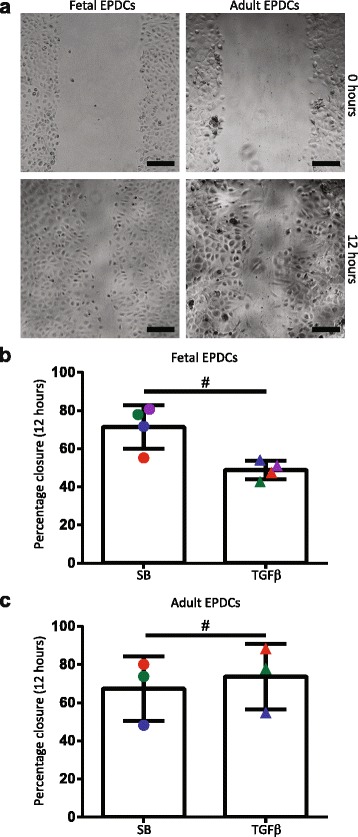
Fetal and adult EPDCs have a different migration ability upon TGFβ stimulation. **a** In a scratch assay, both fetal and adult EPDCs closed the gap as a sheet of cells (scale bar: 100 μm). Quantification of the migration rate after 12 hours showed that, (**b**) upon TGFβ stimulation and thereby partial induction of EMT, the percentage closure decreased for fetal EPDCs (paired *t* test). On the other hand, (**c**) adult EPDCs closed the gap faster upon TGFβ stimulation (paired *t* test). The different colors represent the different donors, for both fetal and adult cultures. ^#^ *p* < 0.05. *EPDCs* epicardial-derived cells, *SB* medium containing SB431542, *TGFβ* transforming growth factor beta

As typical for epithelial cells [31], both fetal and adult EPDCs migrated as a sheet of cells upon closure of the artificial wound (Fig. 4a). Analyzing the percentage of closure after 12 hours showed that in the presence of SB, fetal and adult EPDCs had an equal migratory ability (around 70% closure in Fig. 4b-c). Cellular migration is known to be increased upon the induction of EMT [32]. Therefore, we hypothesized that TGFβ would increase the mobility of EPDCs. As expected, a 2-day stimulation with TGFβ, thereby partially inducing EMT, resulted in an increased migration of adult EPDCs (Fig. 4c). Strikingly, fetal EPDCs responded to TGFβ by decreasing their motility (Fig. 4b). These results confirmed that in vitro EPDCs retained their ability to migrate. Furthermore, it suggests that fetal EPDCs are in a different epithelial activation state, as their migration rate upon TGFβ stimulation resembles the speed of their fully differentiated mesenchymal descendants (as evident from Additional file 1: Figure S5a). Mesenchymal EPDCs are hereafter referred to as spindle-shaped EPDCs (sEPDCs).

The transition into a mesenchymal phenotype has generally been associated with the gain of migratory properties [31]. Interestingly however, fetal and adult sEPDCs displayed a lower migration capacity compared to their epithelial counterparts (Additional file 1: Figure S5a-b). This shows that EMT is not a linear process and cells can acquire migratory properties while still present in their epithelial state, a feature which was previously shown for the mammary epithelium [33].

### Fetal and adult human EPDCs behave differently in tube formation assay

**Fig. 5:**
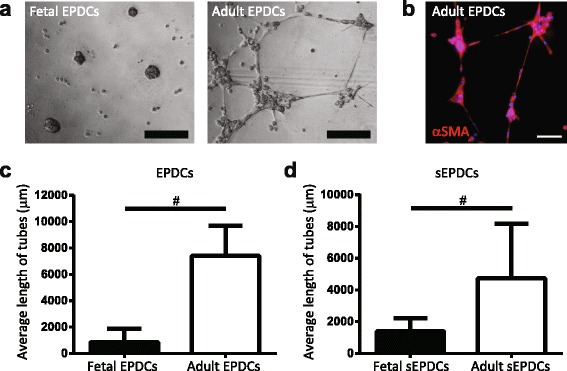
Fetal and adult EPDCs behave differently in tube formation assay. **a** Matrigel-based tube formation assay revealed that fetal EPDCs and sEPDCs failed to form tubes. In contrast, both adult EPDCs and sEPDCs were able to assemble in a network of tube-like structures. **b** Tubes expressed αSMA (scale bars: 100 μm). Tube formation is displayed as average length of tubes for (**c**) fetal and adult EPDCs and (**d**) their fully differentiated mesenchymal counterparts (sEPDCs). ^#^ *p* < 0.05. *EPDCs* epicardial-derived cells, *sEPDCs* spindle-shaped epicardial-derived cells

EPDCs have been suggested to participate in the formation of coronary vessels [4, 5, 21, 34]. Therefore, we assessed the ability of fetal and adult EPDCs to form a network in a Matrigel-based tube formation assay. Interestingly, only adult EPDCs were able to form an αSMA+ tubular network, while fetal EPDCs failed to do so (Fig. 5a–c). A similar trend was observed for mesenchymal EPDCs, showing an increased ability of adult sEPDCs to form tubes compared to fetal sEPDCs (Fig. 5d and Additional file 1: Figure S5c).

Since only adult EPDCs were able to assemble into a tubular network, we questioned whether partial induction of EMT in adult EPDCs would enhance their angiogenic activity, like it increased their migration rate. Therefore, we compared SB-treated cells with their non-treated and TGFβ-stimulated counterparts. Removal of SB and pretreatment with TGFβ increased the vascular potential of adult EPDCs (Additional file 1: Figure S5d).

The inability of fetal cells to form networks suggests that fetal and adult EPDCs have different intrinsic abilities. Interestingly, qRT-PCR analysis for the kinase insert domain receptor (KDR or VEGFR2) revealed that its expression level is higher in adult EPDCs compared to fetal EPDCs (data not shown), and might explain why fetal EPDCs fail to develop tubular structures. Furthermore, taking together our observations from the migration and tube formation assay, our data indicate that partial induction of EMT by TGFβ stimulation increases the ability of adult EPDCs to migrate and form a tubular network. This suggests that adult EPDCs can be present in different stages of epithelial activation and that their cell fate potential is determined by this activation state.

### Human fetal EPDCs are more prone to undergo EMT

**Fig. 6:**
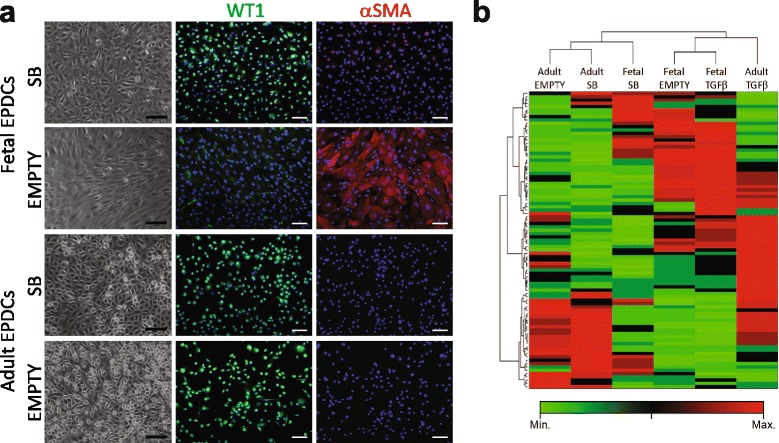
Human fetal EPDCs are more prone to undergo EMT. **a** Upon removal of SB from culture medium, adult EPDCs remained their epithelial character while fetal EPDCs underwent EMT. This was evident by the change in morphology, decrease in nuclear WT1 and increase in αSMA staining (scale bars: 100 μm; staining intensity was corrected to their corresponding SB control). **b** An EMT-PCR array was performed for SB, non- (empty) and TGFβ -stimulated EPDCs and visualized as clustergram using Ward’s linkage. Upon hierarchical clustering, unstimulated adult EPDCs (empty) were closely related to their SB control, while unstimulated fetal EPDCs clustered together with their TGFβ-treated condition. *EPDCs* epicardial-derived cells, *SB* medium containing SB431542, *TGFβ* transforming growth factor beta

Removing cells from their natural in vivo environment into a cell culture situation may potentially result in the induction of EMT. We routinely added the ALK5 kinase inhibitor SB to maintain EPDCs in their epithelial state and prevent the induction of EMT through (endogenous) TGFβ. Thus far, fetal and adult EPDCs cultured under these conditions appeared quite similar with respect to their morphology, expression of epicardial genes, as well as their ability to undergo EMT and migrate. Interestingly, we observed that adult EPDCs relied less on the presence of SB and retained their cobblestone phenotype even when this inhibitor was removed (Fig. 6a). The epithelial nature of fetal EPDCs, on the other hand, was highly dependent on the presence of the ALK5 inhibitor (SB) during culture. Upon removal of SB, fetal EPDCs rapidly underwent spontaneous EMT, demonstrated by their morphological change towards a mesenchymal phenotype (Fig. 6a), withdrawal of WT1 from the nucleus (Additional file 1: Figure S6a) and upregulation of αSMA filaments (Fig. 6a). Furthermore, fetal EPDCs underwent TGFβ-induced EMT at a faster rate, compared to adult EPDCs. In fetal cultures, αSMA+ cells were observed after 24 hours of TGFβ stimulation while for adult cultures this took more than 48 hours (Additional file 1: Figure S6b).

To further verify whether fetal EPDCs indeed respond faster to EMT-related cues, we performed a PCR array for EMT-related genes. Fetal and adult EPDCs were cultured in empty, SB- or TGFβ-containing medium for 4 days. Interestingly, upon hierarchical clustering using Ward’s linkage, non-treated (empty) cells clustered together with SB-treated cells in the adult setting, while the empty condition in fetal cells resembled TGFβ-stimulated cells (Fig. 6b). This pattern was independent of the linkage method used and was consistently observed performing average, single, complete, centroid and median linkage (data not shown). The PCR array shows that fetal EPDCs, upon SB removal, became more closely related to their spindle-shaped descendants in comparison to adult EPDCs. Interestingly, when comparing gene expression between fetal and adult EPDCs, we observed a baseline difference in several EMT-related genes. *CDH1* and annexin A8 (*ANXA8*), genes related to (quiescent) epithelium, are expressed at lower levels in fetal cells (Additional file 1: Figure S6c), while the mesenchymal genes *TCF21* and *VIM* were significantly upregulated in fetal EPDCs (Additional file 1: Figure S6d). Although VIM was highly expressed in fetal EPDCs it did not organize in filaments characteristic for mesenchymal cells (Fig. 3e). Altogether this suggests that fetal EPDCs are more prone to undergo EMT. Furthermore, it shows that fetal epithelial EPDCs, in contrast to adult EPDCs, already have a mesenchymal signature, suggesting that they have the intrinsic ability to undergo EMT.

## Discussion

Since the epicardium envelopes the myocardium, it is relatively easily accessible and it could therefore represent a therapeutic target to facilitate cardiac repair. In this context, EPDCs are an interesting endogenous cell source to modulate scar formation and improve cardiac regeneration [16, 35]. The post-myocardial infarction (MI) response of the adult epicardium is suboptimal compared to the active contribution of the fetal epicardium during development. Furthermore, it is unknown whether activation of the epicardium in the embryo can be extrapolated to the adult setting. Therefore, it is paramount to directly compare fetal and adult EPDCs in order to understand and increase the regenerative and cardiac repair potential of human (adult) EPDCs. However, this has been hampered by the lack of suitable culture protocols.

With the method described in this article, we can now efficiently isolate, expand, and maintain EPDCs derived from adult and fetal human hearts. We have extensively characterized these cells and confirmed their epicardial status. A direct comparison revealed that fetal and adult EPDCs both undergo TGFβ-induced EMT. Although many functional aspects in these cells are similar, we observed several differences that could be related to a different stage of activation. A few protocols for the isolation of EPDCs from mouse and human heart tissue [23, 36–39] as well as from the mouse proepicardium [40] have been reported. However, none of these protocols describe the isolation and subsequent expansion of the fetal and adult human epicardium using the same method and culture conditions. Zhou et al. [39, 41] presented the isolation of mouse EPDCs from both the fetal and adult epicardium using WT1-driven GFP expression to sort EPDCs from a single-cell suspension. Since it is based on reporter gene expression, it is impossible to translate it into a human setting. General isolation based on WT1 antibody binding is not feasible since we found that this protein is also expressed by endothelial cells [42]. More importantly, it is necessary to induce injury to reactivate WT1 expression as it is absent in the adult epicardium [14], which makes it impossible to study adult EPDCs beyond the context of MI.

Assays based on explant outgrowth (including [38, 43, 44]) rely on migration of cells away from the cultured heart tissue. This will promote EMT and onset of mesenchymal differentiation. For the adult mouse heart, it is difficult to separate the epicardium from the myocardium. Therefore isolation of mouse EPDCs relies on explant outgrowth methods. However, outgrowth is not always achieved without the addition of an inducer of migration [45]. For fetal heart tissue, this method results in a limited surface area of cell outgrowth and only a part of the outgrown fetal EPDCs remain in an epithelial state. This is most likely related to our observation that fetal cells are more sensitive to EMT-inducing factors. Importantly, using the outgrowth method to derive fetal and adult cells, but also the WT1-based isolated EPDCs, may represent a selection or subpopulation of epicardial cells.

Protocols have also been developed for the differentiation of EPDCs from human induced pluripotent stem (iPS) cells [46, 47]. However, since it is difficult to fully control the state of the iPS cell-derived EPDCs (fetal- or adult-like), they do not provide the opportunity to compare behavior of the developing and adult epicardium.

In vivo the epicardium is a heterogeneous population, with cells existing in several degrees of activation both during development, as upon reactivation in adulthood [4, 11, 26]. Our protocol enabled us to maintain fetal and adult EPDCs in an epithelial state with an epicardial signature for several passages. However, whilst being quiescent in vivo, the fact that the cells are dividing and express WT1 in culture indicates that they are slightly activated. When comparing EPDCs at baseline, subtle differences between fetal- and adult-derived cells were observed. For instance, the enhanced expression of *ANXA8* and higher levels of *CDH1* in adult EPDCs could imply a more epithelial phenotype. Interestingly, *WT1* mutant epicardium was shown to have increased levels of CDH1, which concurred with reduced epicardial EMT and a lower expression of the mesenchymal marker VIM [1]. Furthermore, the expression of *TCF21,* which has been shown to play a role in cardiac fibroblast specification [11], was upregulated in fetal EPDCs suggesting that these cells are already partly committed to the mesenchymal lineage.

The distinction between fetal and adult cells became more obvious in the functional assays. Both fetal and adult EPDCs migrated at similar speeds at baseline. TGFβ has been shown to activate an epicardial motile gene program [48]. Indeed, short-term TGFβ stimulation increased the migration of adult EPDCs. In contrast, in fetal EPDCs addition of TGFβ resulted in a significantly delayed migration, whereby they resembled the migration speed of both fetal and adult sEPDCs. In addition to increased migration, induction of EMT with TGFβ stimulation increased the tube formation ability of adult EPDCs. Altogether, this suggests that fetal and adult EPDCs are in a different stage of epithelial activation and shows that the EPDC cell fate potential is determined by its state of activation.

The concept that fetal EPDCs are in a different state of activation, was strengthened by our observation that fetal EPDCs respond faster to external cues like TGFβ stimulation. Furthermore, fetal EPDCs are more dependent on SB for their cobblestone morphology than their adult counterparts. This could indicate that fetal cells have a higher intrinsic and/or autocrine ability to undergo EMT. This hypothesis is similar to what is seen in embryonic endothelial cells which partly via autocrine signaling underwent mesenchymal transformation, thereby contributing to cushion formation [49].

Another potential explanation for this spontaneous EMT could be that fetal EPDCs are mostly ventricular while adult EPDCs are derived from the atrial epicardium. Using an explant culture method, Risebro et al. [50] showed that embryonic ventricular EPDCs spontaneously undergo EMT while atrium-derived embryonic EPDC remain their epithelial phenotype. However, we were able to derive epithelial EPDCs from both the developing atrium as well as the ventricle (data not shown). This suggests that, at the developmental stages we have studied, epicardial activation is independent of the anatomic location of the epicardium along the cardiac outline.

Taken together, our data suggest that adult EPDCs exist in culture in an epithelial state, and require more time and external cues to obtain mesenchymal properties. Fetal EPDCs are more mesenchymal in vitro and therefore likely much more prone to undergo EMT. The question remains whether adult EPDCs have lost this feature, and if it could be reestablished provided the right cues are present.

## Conclusions

In conclusion, our primary EPDC cultures will provide important knowledge regarding the human epicardial activation process and epicardial behavior during development and adult life. It provides the opportunity to identify factors that are involved in EMT, migration, and differentiation of human EPDCs. Furthermore, it will give us insight into their signaling pathway sensitivity, paracrine signals, and differentiation capabilities. More importantly, this model allows the direct comparison between fetal and adult EPDCs, which will be important given the embryonic activation response of the epicardium following injury. As such, it may enable us to direct the human adult EPDC fate in favor of cardiac regeneration in the future.

### Clinical relevance

Cardiac repair can be achieved by using inherent repair mechanisms. Given its pivotal role during development and cardiac injury, the epicardium is gaining recognition as an endogenous cell source for cardiac repair. EPDCs could interfere with several aspects of infarct healing. First, the paracrine factors they secrete could prevent or decrease the loss of contractile units. Second, EPDCs play a role in scar formation and therefore, modulating their behavior could ameliorate cardiac function. And third, EPDCs could potentially contribute to vascularization and myogenesis, both needed for cardiac recovery. To understand the full potential of adult EPDCs, we require a detailed understanding of the abilities of adult EPDCs to contribute to cardiac repair, including the potential these cells once had during development.

## Additional file

Additional file 1: Figure S1. Isolation of human epicardium. (a) For isolation of EPDCs, the epicardium was removed from the underlying myocardium. (b) The isolated epicardial layer was positive for *WT1* and negative for the myocardial markers *cTnT* and *βMHC*. In addition, the epicardial layer expressed, compared to myocardial tissue, low levels of the smooth muscle marker *αSMA*. This data was representative for three independent isolations, which all together showed the purity of the isolated epicardial layer. **Figure S2.** Human fetal and adult EPDCs express epicardium-related markers. (a) Summary of qRT-PCR analysis, indicating expression levels of genes reportedly expressed in the epicardium or EPDCs, showed that both fetal and adult EPDCs were positive for genes investigated. (b) WT1, (c) TBX18, and (d) TCF21 protein were present within the nucleus of both EPDC populations. **Figure S3.** Cultured human fetal and adult EPDCs do not display markers attributed to other heart-resident cell types. Gene expression was determined by qRT-PCR using several heart-related markers for (a-b) cardiac fibroblasts, (c) endocardium, (d) endothelial cells, (e) cardiac progenitor cells, (f) hematopoietic cells, and (g) mature cardiomyocytes. All data was shown as fold increase compared to their corresponding control cell type or tissue (*N.D.*: not detected). **Figure S4.** Fetal and adult EPDCs undergo EMT upon TGFβ stimulation. Validation of EMT was determined by qRT-PCR showing the downregulation of the epithelial genes *ALDH1A2* and *CDH1* in (a) fetal and (b) adult EPDCs. In addition, *POSTN* and *FN1* were upregulated upon TGFβ stimulation in both (c) fetal and (d) adult EPDCs (data are representative of at least three independent EMT experiments). **Figure S5.** Migration and tube formation capacity of human EPDCs and sEPDCs. Scratch assays revealed that TGFβ stimulation decreased the migration rate of both (a) fetal and (b) adult sEPDCs. Furthermore, the *dotted line*, representing the migration rate of fetal and adult EPDCs, suggested that sEPDCs have an equal or even decreased ability to migrate. In the tube formation assay, comparing fetal and adult mesenchymal EPDCs, (c) adult sEPDCs had an increased ability to assemble into a closed tubular network. (d) Matrigel-based tube formation assays revealed that upon partial induction of EMT (untreated or TGFβ-treated) adult EPDCs formed more tubes. ^#^ *p* < 0.05. **Figure S6.** Fetal EPDCs are more prone to undergo EMT. (a) Upon removal of SB for a period of 4 days, WT1 protein was only withdrawn from the nucleus of fetal EPDCs. (b) Upon TGFβ stimulation, fetal EPDCs underwent EMT at a faster rate. This was shown by the occurrence of αSMA+ fibers in fetal cultures already after 24 hours (day 1) of stimulation (scale bar: 50 μm). (c) The epithelial-related factors, *CDH1* and *ANXA8*, were increased in adult EPDCs, (d) while the mesenchymal genes *TCF21* and *VIM* were upregulated in fetal EPDCs at baseline. ^#^ *p* < 0.05. (PDF 491 kb)

## Abbreviations

EMT: epithelial-to-mesenchymal transition
EPDCs: epicardial-derived cells
FCS: fetal calf serum
iPS cell: induced pluripotent stem cell
MI: myocardial infarction
SB: SB431542 (inhibitor of the TGFβ receptor ALK5)
sEPDCs: spindle-shaped epicardial-derived cells
TGFβ: transforming growth factor beta
