# Supplementary figures and images for: Human fetal and adult epicardial-derived cells: a novel model to study their activation
Source: Stem Cell Res Ther. 2016 Nov 29;7:174. doi: 10.1186/s13287-016-0434-9 (PMC5129650; doi:10.1186/s13287-016-0434-9)

**Figure S1**

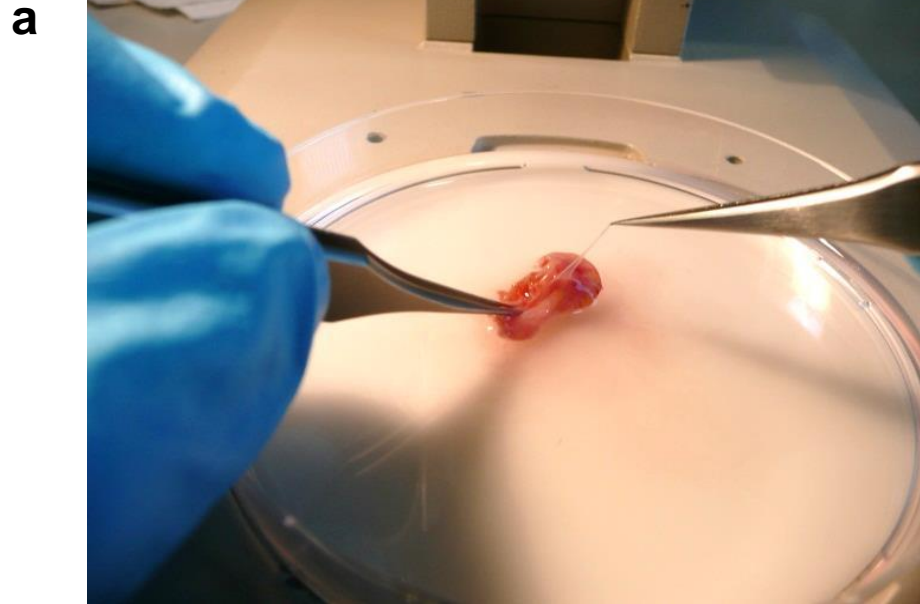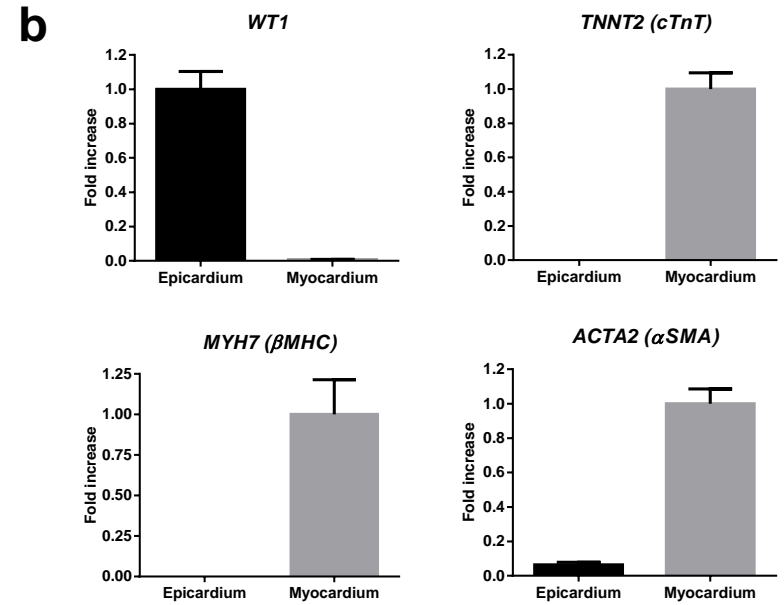

**Figure S2**

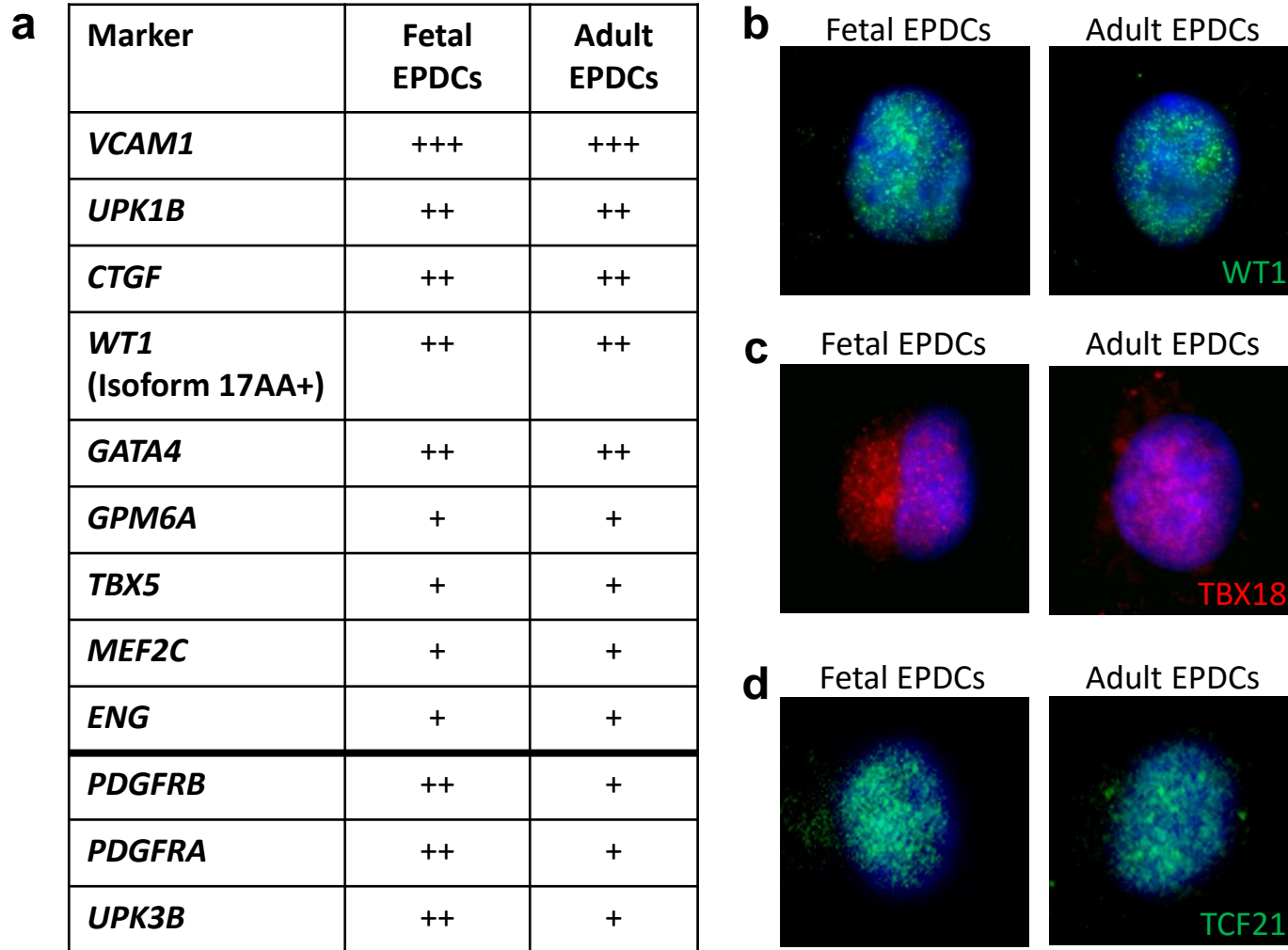

Figure S3

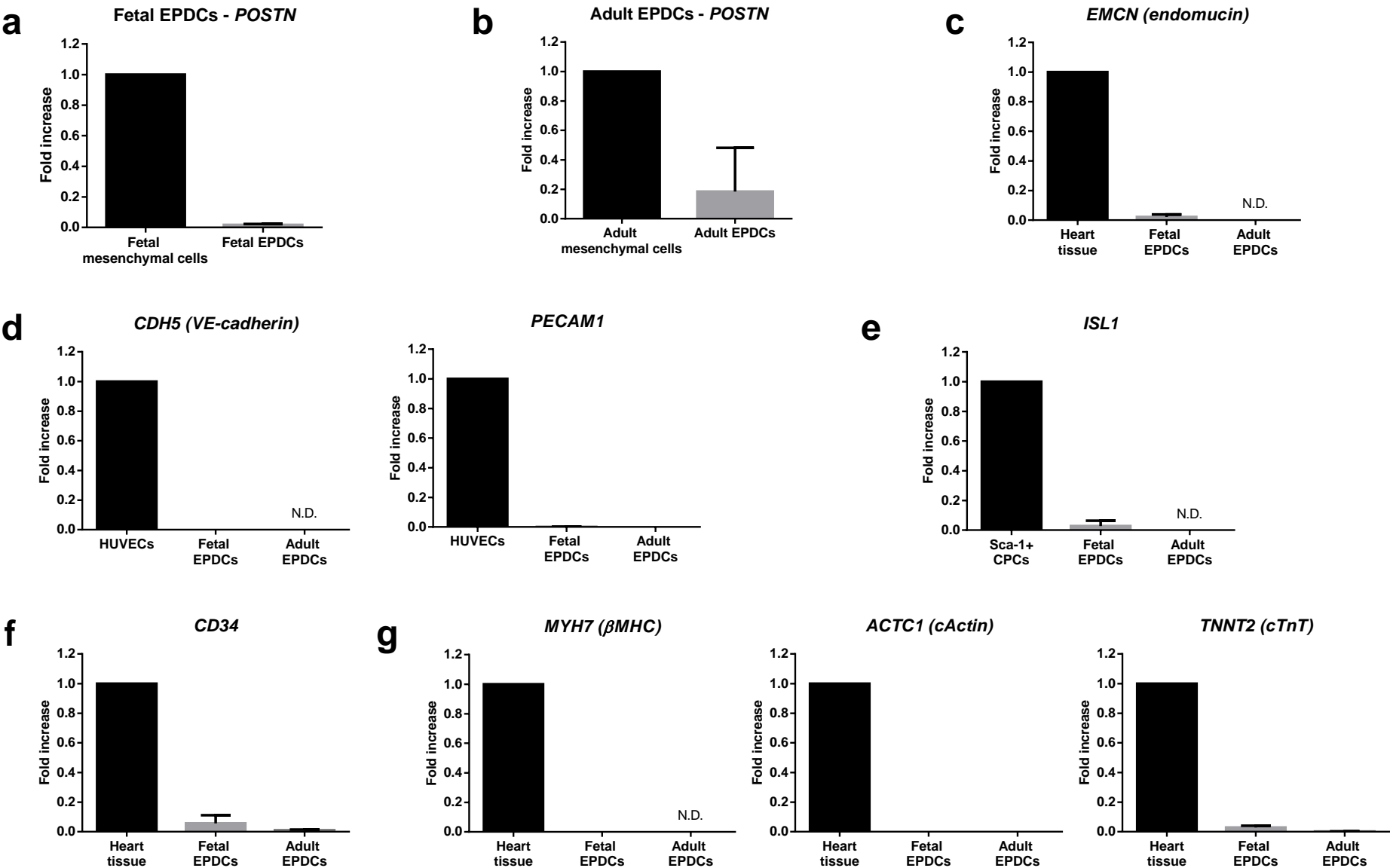

Figure S4

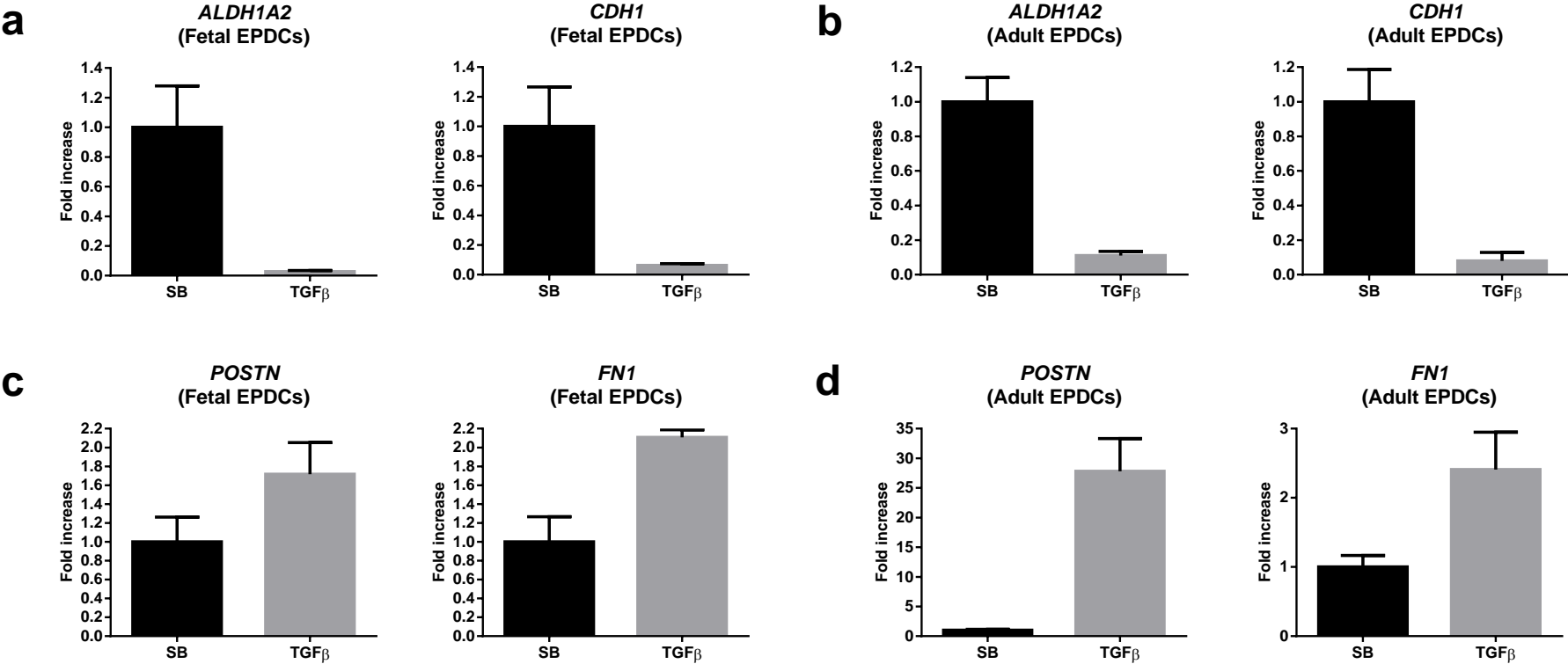

**Figure S5**

**a**

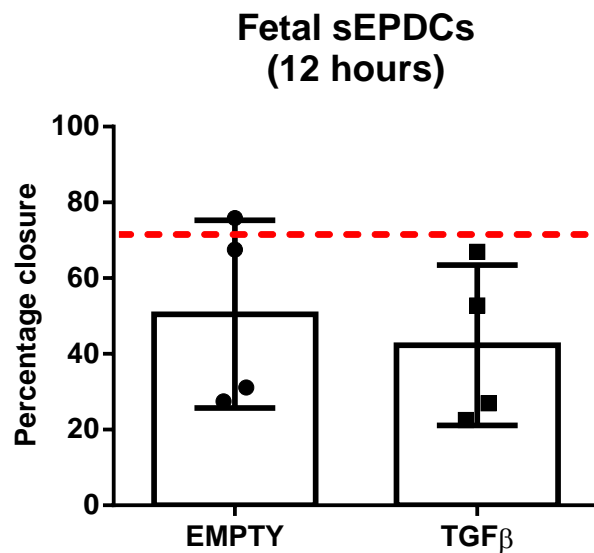

**b**

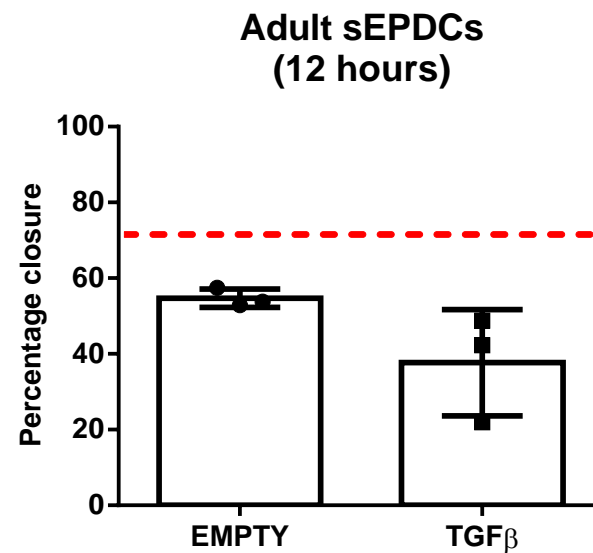

**c**

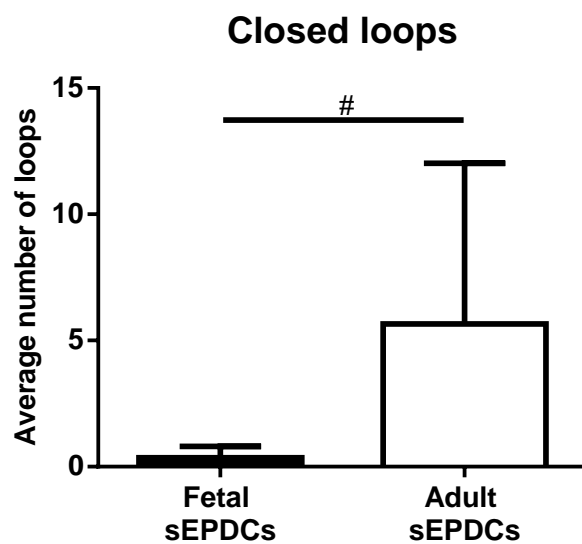

**d**

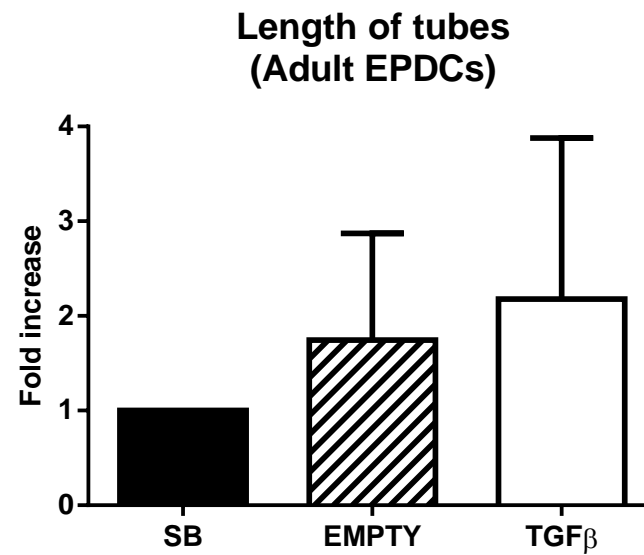

Figure S6

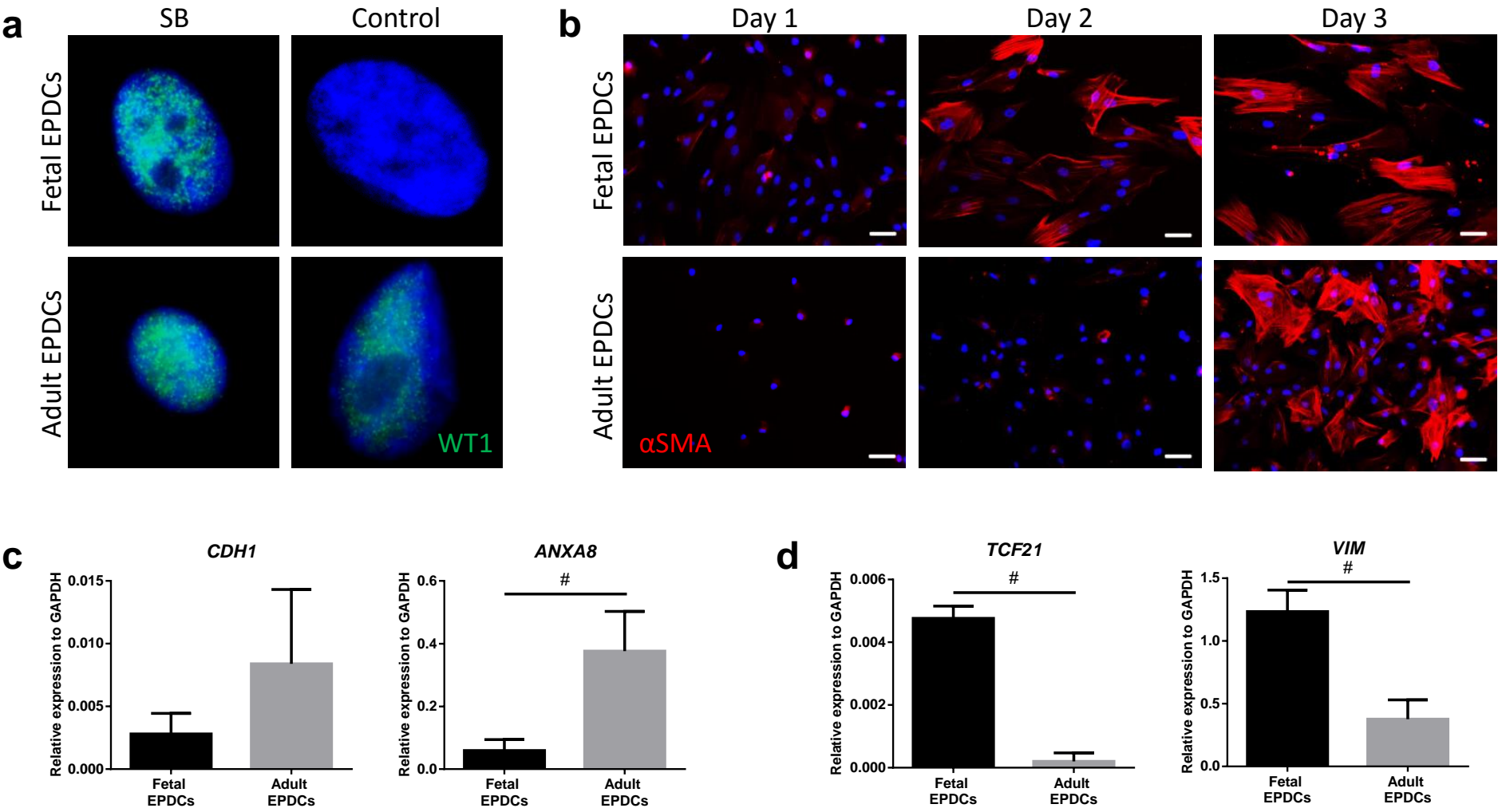

Supplement: Additional file 1: Figure S1. — Isolation of human epicardium. (a) For isolation of EPDCs, the epicardium was removed from the underlying myocardium. (b) The isolated epicardial layer was positive for WT1 and negative for the myocardial markers cTnT and βMHC. In addition, the epicardial layer expressed, compared to myocardial tissue, low levels of the smooth muscle marker αSMA. This data was representative for three independent isolations, which all together showed the purity of the isolated epicardial layer. Figure S2. Human fetal and adult EPDCs express epicardium-related markers. (a) Summary of qRT-PCR analysis, indicating expression levels of genes reportedly expressed in the epicardium or EPDCs, showed that both fetal and adult EPDCs were positive for genes investigated. (b) WT1, (c) TBX18, and (d) TCF21 protein were present within the nucleus of both EPDC populations. Figure S3. Cultured human fetal and adult EPDCs do not display markers attributed to other heart-resident cell types. Gene expression was determined by qRT-PCR using several heart-related markers for (a-b) cardiac fibroblasts, (c) endocardium, (d) endothelial cells, (e) cardiac progenitor cells, (f) hematopoietic cells, and (g) mature cardiomyocytes. All data was shown as fold increase compared to their corresponding control cell type or tissue (N.D.: not detected). Figure S4. Fetal and adult EPDCs undergo EMT upon TGFβ stimulation. Validation of EMT was determined by qRT-PCR showing the downregulation of the epithelial genes ALDH1A2 and CDH1 in (a) fetal and (b) adult EPDCs. In addition, POSTN and FN1 were upregulated upon TGFβ stimulation in both (c) fetal and (d) adult EPDCs (data are representative of at least three independent EMT experiments). Figure S5. Migration and tube formation capacity of human EPDCs and sEPDCs. Scratch assays revealed that TGFβ stimulation decreased the migration rate of both (a) fetal and (b) adult sEPDCs. Furthermore, the dotted line, representing the migration rate of fetal and adult EPDCs, sugg [file 13287_2016_434_MOESM1_ESM.pdf]
